# *Lutzomyia migonei* is a permissive vector competent for *Leishmania infantum*

**DOI:** 10.1186/s13071-016-1444-2

**Published:** 2016-03-17

**Authors:** Vanessa Cristina Fitipaldi Veloso Guimarães, Katerina Pruzinova, Jovana Sadlova, Vera Volfova, Jitka Myskova, Sinval Pinto Brandão Filho, Petr Volf

**Affiliations:** Department of Immunology, Centro de Pesquisas Aggeu Magalhães, Fundação Oswaldo Cruz (Fiocruz), Recife, Pernambuco Brazil; Department of Parasitology, Faculty of Science, Charles University, Prague, Czech Republic

**Keywords:** *Lutzomyia migonei*, *Leishmania infantum*, Vector competence

## Abstract

**Background:**

*Leishmania infantum* is the most widespread etiological agent of visceral leishmaniasis (VL) in the world, with significant mortality rates in human cases. In Latin America, this parasite is primarily transmitted by *Lutzomyia longipalpis*, but the role of *Lutzomyia migonei* as a potential vector for this protozoan has been discussed. Laboratory and field investigations have contributed to this hypothesis; however, proof of the vector competence of *L. migonei* has not yet been provided. In this study, we evaluate for the first time the susceptibility of *L. migonei* to *L. infantum*.

**Methods:**

Females of laboratory-reared *L. migonei* were fed through a chick-skin membrane on rabbit blood containing *L. infantum* promastigotes, dissected at 1, 5 and 8 days post-infection (PI) and checked microscopically for the presence, intensity and localisation of *Leishmania* infections. In addition, morphometric analysis of *L. infantum* promastigotes was performed.

**Results:**

High infection rates of both *L. infantum* strains tested were observed in *L. migonei*, with colonisation of the stomodeal valve already on day 5 PI. At the late-stage infection, most *L. migonei* females had their cardia and stomodeal valve colonised by high numbers of parasites, and no significant differences were found compared to the development in *L. longipalpis.* Metacyclic forms were found in all parasite-vector combinations since day 5 PI.

**Conclusions:**

We propose that *Lutzomyia migonei* belongs to sand fly species permissive to various *Leishmania* spp. Here we demonstrate that *L. migonei* is highly susceptible to the development of *L. infantum*. This, together with its known anthropophily, abundance in VL foci and natural infection by *L. infantum*, constitute important evidence that *L. migonei* is another vector of this parasite in Latin America.

## Background

Visceral leishmaniasis (VL) caused by protozoa of the genus *Leishmania* (Kinetoplastida: Trypanosomatidae) is a vector-borne neglected disease transmitted by phlebotomine sand flies (Diptera: Psychodidae) [1]. *Leishmania infantum* (syn. *Leishmania chagasi*) is the most widespread etiological agent of VL in the world, including Latin America, with significant mortality rates in human cases [2]. Approximately 56 phlebotomine sand fly species are supposed, or have been proved, to be involved in the transmission of *Leishmania* spp. in the Americas [1]. Unfortunately, control strategies against vectors as well as reservoirs of leishmaniasis have been ineffective [3].

In Latin America, *L. infantum* is primarily transmitted by *Lutzomyia longipalpis* [4]. This sand fly species fulfils all criteria of a proven vector, which includes the ability of the insect to support the development of the parasite during and after bloodmeal digestion, as well as to transmit them to a susceptible host [5]. On the other hand, information about the vector competence of other *Lutzomyia* spp. to *L. infantum* is lacking, despite field data suggesting their possible involvement in the circulation of this parasite.

The vectorial role of *Lutzomyia migonei* has been discussed in areas with a record of human and canine cases of VL but where the proven vector is absent [6–9]. In a study conducted in southeast Brazil, the absence of *L. longipalpis* in six endemic areas provided circumstantial evidence for the participation of *L. migonei* in the transmission of *L. infantum* [6], and more recent studies have reinforced this hypothesis [7, 9]. In VL foci in northeast Brazil and northeast Argentina, *L. migonei* was reported as a predominant species associated with human and canine cases in the peridomicile environment [7, 9]. Additionally, some investigations have reported the detection of *L. infantum* DNA in this sand fly species [8, 10], highlighting the need for additional evidence in order to confirm *L. migonei* as a vector of *L. infantum*.

*Lutzomyia migonei* is widespread in South America, including Brazil, and shows adaptability to modified environments, being found in human dwellings and animal shelters [11, 12]. This sand fly species displays anthropophilic behaviour and has opportunistic feeding habits, including on dogs, chickens, equines and wild animals [11, 13]. Furthermore, *L. migonei* has been implicated as a vector of *L*. (*V*.) *braziliensis*, the etiological agent of cutaneous leishmaniasis in different Brazilian regions [11, 14, 15].

Despite the epidemiological and behavioural characteristics that indicate the participation of *L. migonei* in the transmission cycle of *L. infantum*, no studies have assessed the ability of this species to support the full development of the parasite. From this perspective, therefore, we evaluate for the first time the susceptibility of freshly colonised specimens of *L. migonei* to experimental infection by *L. infantum*.

## Methods

### Sand fly colonies and *Leishmania* strains

A colony of *Lutzomyia migonei* was established at Charles University in Prague from specimens captured in Baturité municipality, Ceará state, northeast Brazil (04°19′41″S, 38°53′05″W). An already-established laboratory colony of *Lutzomyia longipalpis* (from Jacobina, Brazil) with well-known susceptibility to *L. infantum* [16–18] was used as a control. Both colonies were maintained under standard conditions as previously described [19].

A viscerotropic *Leishmania infantum* strain (MHOM/BR/76/M4192) [20] and dermotropic *L. infantum* strain (ITOB/TR/2005/CUK3) [21] were maintained at 23 °C on Medium 199 (Sigma) supplemented with 10 % foetal calf serum (Gibco), 1 % BME vitamins (Sigma), 2 % human urine and 250 μg/ml amikin (Amikin, Bristol-Myers Squibb).

### Experimental infections of sand flies

Sand fly females (2 to 6 days old) were fed through a chick-skin membrane on heat-inactivated rabbit blood containing 10^6^ promastigotes/ml. The experiments were conducted with three sand fly-*Leishmania* combinations: *L. migonei*-CUK3, *L. migonei-*M4192, and *L. longipalpis*-M4192. A fourth combination, the CUK3 strain in *L. longipalpis*, was previously studied in detail [18]. Engorged females were separated and maintained in the same conditions as the colony and dissected on days 1, 5 and 8 post-infection (PI). Individual guts were placed into a drop of saline and examined microscopically for the localisation and intensity of *Leishmania* infections. Parasite loads were graded according to Myskova et al. [22] as light (<100 parasites per gut), moderate (100 to 1000 parasites per gut) and heavy (>1000 parasites per gut). The experiment was repeated five times. Data were evaluated statistically by means of the Fisher’s exact or Chi-square (*χ*^2^) tests using SPSS statistics 23 software.

### Morphometry of parasites

Smears from midguts of *L. migonei* and *L. longipalpis* infected with *L. infantum* on days 5 and 8 PI were fixed with methanol, stained with Giemsa, examined under a light microscope with an oil-immersion objective and photographed with an Olympus D70 camera. Body length, body width and flagellar length of 240 randomly selected promastigotes from six females/smears were measured for each sand fly species and time interval using Image-J software. The morphological forms were distinguished according to Walters [23] and Cihakova & Volf [24]: (i) short nectomonads: body length < 14 μm and flagellar length < 2 times body length; (ii) long nectomonads: body length ≥14 μm; (iii) metacyclic promastigotes: body length < 14 μm and flagellar length ≥2 times body length. Data were evaluated statistically by analysis of variance using SPSS statistics 23 software.

### Ethical approval

Animals were maintained and handled in the animal facility of Charles University in Prague in accordance with institutional guidelines and Czech legislation (Act No. 246/1992 and 359/2012 coll. on Protection of Animals against Cruelty in present statutes at large), which complies with all relevant EU guidelines for experimental animals. All experiments were approved by the Committee on the Ethics of Laboratory Experiments of the Charles University in Prague and were performed under the Certificate of Competency (Registration Number: CZ 03069).

## Results

### Susceptibility of *Lutzomyia migonei* and *L. longipalpis* to *Leishmania infantum*

**Fig. 1:**
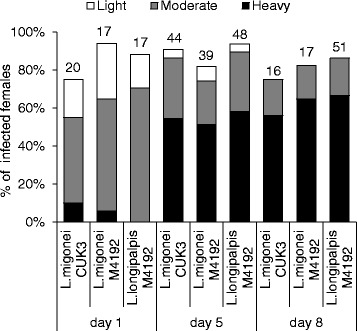
Development of *L. infantum* in *Lutzomyia *spp. Rates and intensities of infections in *Lutzomyia migonei* and *Lutzomyia longipalpis* were evaluated microscopically on days 1, 5 and 8 PI, and were classified into three categories: light (<100 parasites/gut), moderate (100–1000 parasites/gut), or heavy (>1000 parasites/gut). Numbers of dissected females are shown above the bars

The development of two strains of *L. infantum* was studied in *L. migonei* and *L. longipalpis* from day 1 to day 8 PI. On day 1 PI, midgut infection rates were high in all parasite-vector combination (75–95 %), with parasites located in the endoperitrophic space within the bloodmeal surrounded by the peritrophic matrix (PM). The parasites developed similarly and no significant differences were found in infection rates (*χ*^*2*^ = 2.84, *df* = 2, *P* = 0.24) or intensities of infection (*χ*^*2*^ = 5.7, *df* = 6, *P* = 0.45; Fig. 1).

**Fig. 2:**
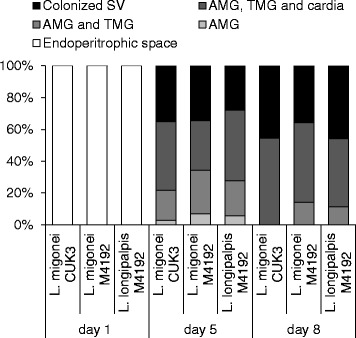
Localisation of *L. infantum* in *Lutzomyia *spp. Localisation of infections in *Lutzomyia migonei* and *Lutzomyia longipalpis* was evaluated microscopically on days 1, 5 and 8 PI. Abdominal midgut (AMG), thoracic midgut (TMG) and stomodeal valve (SV)

On day 5 PI, when the bloodmeal was digested and remnants defecated in both species, infection rates remained high (above 80 %) and most infections were heavy in all parasite-vector combinations evaluated. Parasites migrated anteriorly to colonise the thoracic midgut and cardia region in 43 % of *L. migonei*-CUK3, 31 % of *L. migonei*-M4192 and 44 % of *L. longipalpis*-M4192. The colonisation of the stomodeal valve was observed most frequently in *L. migonei*-CUK3 (35 % of infected females), followed by *L. migonei*-M4192 (34 % of infected females) and *L. longipalpis*-M4192 (27 % of infected females). Statistical analysis did not show any significant differences in the localisation of infections between the experimental groups (*χ*^*2*^ = 2.3, *df* = 6, *P* = 0.88; Fig. 2).

**Fig. 3:**
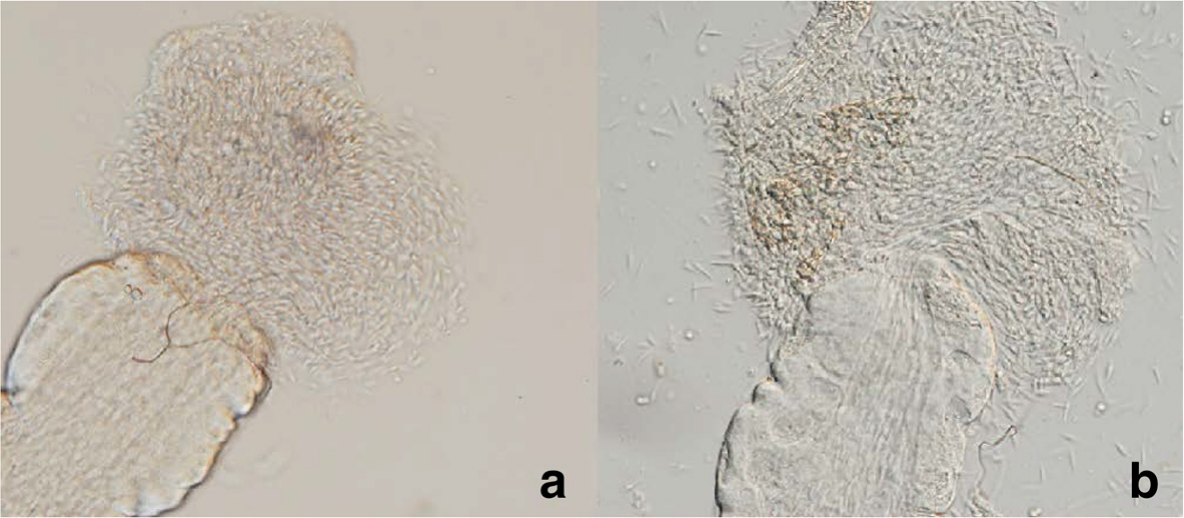
Thoracic midgut with cardia section and stomodeal valve of *Lutzomyia migonei* females. **a** Infection of *L. infantum* CUK3 on day 5 PI. **b** Infection of *L. infantum* M4192 on day 8 PI

On day 8 PI, both *L. infantum* strains continued to develop successfully in *L. migonei* and *L. longipalpis*. In all parasite-vector combinations, infection rates were above 75 % and parasites developed heavy late-stage infections in the majority of infected females. The colonisation of the stomodeal valve was observed in 45 % of *L. migonei*-CUK3 and *L. longipalpis*-M4192 and 35 % of *L. migonei*-M4192. Similarly, no significant differences were observed in the localisation of infections (*χ*^*2*^ = 1.9, *df* = 4, *P* = 0.74). Light microscopy showed a mass of *Leishmania* promastigotes in the cardia region and attached to the stomodeal valve of females. In dissected midguts, this parasite mass accompanied by promastigote-secretory gel erupted from the valve (Fig. 3).

### Morphometric analysis of *L. infantum* promastigotes in *L. migonei* and *L. longipalpis*

Morphological analysis was performed on *L. infantum* parasites from *L. migonei* and *L. longipalpis* on days 5 and 8 PI. Differences in all parameters analysed, i.e. body length, body width and flagellar length, from all parasite-vector combinations are summarized in Table 1.

**Table 1:** Dimensions of the morphological forms of *L. infantum* described in Fig. 4 during development in *L. migonei* and *L. longipalpis* on days 5 and 8 PI

				Body length		Body width		Flagellar length	
Day PI	Parasite-vector combination	Morphological form	*n*	Mean (SD) (μm)	Range (μm)	Mean (SD) (μm)	Range (μm)	Mean (SD) (μm)	Range (μm)
5	*L. migonei*-CUK3	LN	78	15.8 (1.4)	14.0–19.1	2.4 (0.4)	1.6–3.7	17.6 (3.5)	7.0–26.1
		SN	155	10.4 (2.0)	5.9–13.8	2.3 (0.5)	1.2–3.7	13.7 (3.4)	5.5–24.2
		MP	7	8.2 (1.2)	6.4–9.9	2.6 (0.5)	1.9–3.6	19.0 (4.5)	13.8–24.8
		Total	240	12.1 (3.1)	5.9–19.1	2.4 (0.5)	1.2–3.7	15.1 (3.9)	5.5–26.1
	*L. migonei*-M4192	LN	125	16.1 (1.5)	14.0–20.9	2.6 (0.5)	1.7–4.2	18.1 (4.5)	6.1–31.7
		SN	107	11.7 (1.7)	6.8–13.9	2.6 (0.6)	1.4–5.9	15.7 (4.8)	6.8–27.7
		MP	8	8.5 (0.6)	7.7–9.8	2.7 (1.1)	1.4–5.2	20.3 (3.5)	15.4–25.1
		Total	240	13.9 (2.8)	6.8–20.9	2.6 (0.6)	1.4–5.9	17.1 (4.8)	6.1–31.7
	*L. longipalpis-*M4192	LN	171	17.5 (3.0)	14.0–26.8	2.4 (0.5)	1.0–3.8	22.0 (6.0)	4.5–36.8
		SN	68	11.2 (1.8)	7.0–13.9	2.4 (0.5)	1.1–3.8	14.8 (4.5)	7.9–24.1
		MP	1	13.7		2.3		28.0	
		Total	240	15.7 (3.9)	7.0–26.8	2.4 (0.5)	1.0–3.8	20.0 (6.5)	4.5–36.8
8	*L. migonei*-CUK3	LN	48	16.0 (2.0)	14.0–22.1	2.4 (0.5)	1.5–3.8	16.2 (2.7)	9.6–21.7
		SN	180	10.2 (2.0)	5.0–13.9	2.1 (0.6)	1.0–4.4	13.5 (3.2)	5.4–24.1
		MP	12	6.6 (1.4)	3.6–8.8	1.8 (0.5)	1.1–3.0	15.7 (2.9)	8.7–19.2
		Total	240	11.2 (3.2)	3.6–22.1	2.1 (0.6)	1.0–4.4	14.2 (3.3)	5.4–24.1
	*L. migonei*-M4192	LN	35	15.4 (1.1)	14.0–18.1	2.2 (0.4)	1.4–3.3	16.1 (4.1)	8.0–24.0
		SN	187	9.8 (2.1)	5.4–13.9	2.4 (0.5)	1.1–4.1	13.4 (3.5)	3.7–24.0
		MP	18	6.9 (1.2)	5.0–10.3	2.1 (0.7)	1.2–3.8	16.4 (2.0)	13.5–22.2
		Total	240	10.4 (2.9)	5.0–18.1	2.3 (0.5)	1.1–4.1	14.0 (3.7)	3.7–24.0
	*L. longipalpis-*M4192	LN	56	15.9 (1.3)	14.0–19.2	2.2 (0.4)	1.6–3.4	18.1 (4.1)	8.2–28.4
		SN	152	10.5 (1.9)	6.4–13.9	2.2 (0.6)	1.1–4.6	14.4 (3.1)	7.3–22.8
		MP	32	7.3 (1.3)	5.1–12.2	2.1 (0.8)	1.1–5.6	16.4 (2.5)	12.7–26.3
		Total	240	11.3 (3.2)	5.1–19.2	2.2 (0.8)	1.1–5.6	15.5 (3.6)	7.3–28.4

*LN* Long nectomonads, *SP* short promastigotes *MP* metacyclic promastigotes

Promastigotes from gut smears were measured under light microscopy with an oil-immersion objective

**Fig. 4:**
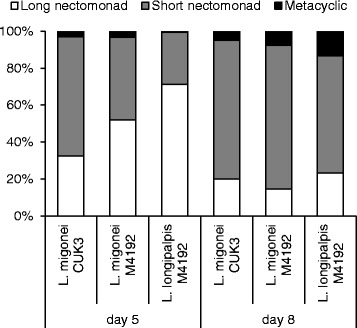
Morphological forms of *L. infantum* during development in *Lutzomyia *spp. Morphological forms of *Leishmania* parasites in *Lutzomyia migonei* and *Lutzomyia longipalpis* were evaluated microscopically on days 5 and 8 PI. Differences between the morphological forms were significant on days 5 (*χ* ^*2*^ = 74.5, *df* = 4, *P* < 0.001) and 8 (*χ* ^*2*^ = 19, *df* = 4, *P* = 0.001) PI

On day 5 PI, slight differences were found between the development of various parasite strains: in *L. migonei* (52 %) and *L. longipalpis* (71 %) infected by *L. infantum* M4192 the majority of parasites were long nectomonads, while in *L. migonei* infected by *L. infantum* CUK3, 64 % of the parasites were short nectomonads. The metacyclic forms were found in all parasite-vector combinations but in low numbers, from 1 to 3 %. Differences in promastigote stages between *Leishmania*-vector combinations were statistically significant (*χ*^*2*^ = 74.5, *df* = 4, *P* < 0.001; Fig. 4).

On day 8 PI, parasites developed similarly and the percentage of metacyclic forms increased in all three combinations studied. There were no significant differences in the morphological forms of *L. infantum* M4192 and CUK3 in *L. migonei* females (*χ*^*2*^ = 3.3, *df* = 2, *P* = 0.18). As expected, the percentage of long nectomonads decreased, while the short nectomonad forms increased to 78 % and 75 %, respectively. The metacyclic forms were found in 7.5 % and 5 % for M4192 and CUK3, respectively. In *L. longipalpis*, the proportion of morphological forms differed: the proportion of short nectomonads was lower while metacyclic forms were more numerous (23 % long nectomonads, 63 % short nectomonads and 13 % metacyclic forms; *χ*^*2*^ = 19.0, *df* = 4, *P* = 0.001).

## Discussion

Although *Leishmania-*vector interaction studies are crucial for understanding the parasite transmission and epidemiology of leishmaniases, only a limited number of New World sand fly species have been evaluated experimentally for susceptibility to *Leishmania* spp. The development of *L. infantum* has been studied repeatedly in *L. longipalpis* (e.g. [16, 18, 25, 26], but information about the vector competence of other species of *Lutzomyia* to this parasite remain unclear.

Here we show for the first time the high susceptibility of *L. migonei* to viscerotropic (M4192) and dermotropic (CUK3) strains of *L. infantum*. The parasites survived bloodmeal digestion, avoided expulsion during the defecation process, established late-stage infections in the midgut, and colonised the thoracic midgut and stomodeal valve of sand fly females, which is the prerequisite for successful transmission by bite [26–28]. Furthermore, the development of both *L. infantum* strains in *L. migonei* was similar, and no significant differences were found in comparison with the development in *L. longipalpis*. The parasites developed relatively quickly, with the presence of metacyclic promastigotes and colonisation of the stomodeal valve already on day 5 PI. At the late-stage infection (on day 8 PI), high numbers of the parasites colonised the midgut cardia and stomodeal valve in females of both species.

In *L. migonei*, the dermotropic CUK3 strain morphologically transformed slightly faster than the viscerotropic M4192 strain, with a slightly higher percentage of short nectomonads in CUK3 observed on day 5 PI. However, similar numbers of metacyclic promastigotes were found in both parasite-vector combinations. The percentage of metacyclic forms (5–7.5 %) observed in *L. migonei* on day 8 is comparable to the percentage of metacyclics previously reported for the *L. infantum-L. longipalpis* combination [29, 30].

According to Maroli et al. [1], incriminating a sand fly species as a vector must be based on an epidemiological context and there must exist a relationship between the geographical distribution of the vector and the record of human cases of the disease, as well as proof of the anthropophily of the species and the vector’s ability to support infection with the same parasite species occurring in humans. *Lutzomyia migonei* is well-known for its opportunistic feeding habits and anthropophilic behaviour [11, 13] and has repeatedly been found to be naturally infected by *L. infantum* in Brazil [8] and Argentina [10]. In this context, our demonstration of the high susceptibility of *L. migonei* to infection by *L. infantum* M4192 is crucial for the epidemiology of VL in Latin America, since this *L. infantum* strain was isolated in northeast Brazil from a patient with VL.

Sand flies have been classified as permissive or specific vectors on account of their capability to support the development of either a wide or limited spectrum of *Leishmania* spp., respectively [27, 28, 31]. Since it has been reported that *L. migonei* supports the development of other species of *Leishmania*, namely *L. braziliensis* and *L. amazonensis* [32], we propose that this sand fly species is a permissive vector.

## Conclusions

This study provides experimental evidence that *L. infantum* develops late-stage infections in *L. migonei*. These results contribute to a better understanding of the role of this sand fly species in the epidemiology of VL caused by *L. infantum*, and allows estimations of the risk of new VL foci in South America. Furthermore, this knowledge is critical for the development of more specific and effective control strategies in endemic areas.
